# Total Genotype Score Modelling of Polygenic Endurance-Power Profiles in Lithuanian Elite Athletes

**DOI:** 10.3390/genes12071067

**Published:** 2021-07-13

**Authors:** Erinija Pranckeviciene, Valentina Gineviciene, Audrone Jakaitiene, Laimonas Januska, Algirdas Utkus

**Affiliations:** Department of Human and Medical Genetics, Biomedical Science Institute, Vilnius University, 01513 Vilnius, Lithuania; valentina.gineviciene@mf.vu.lt (V.G.); audrone.jakaitiene@mf.vu.lt (A.J.); laimonas.januska@mf.vu.lt (L.J.); algirdas.utkus@mf.vu.lt (A.U.)

**Keywords:** human athletic performance, Lithuanian athletes, polygenic profile, total genotype score, TGS, logistic regression

## Abstract

Total genotype score (TGS) reflects additive effect of genotypes on predicting a complex trait such as athletic performance. Scores assigned to genotypes in the TGS should represent an extent of the genotype’s predisposition to the trait. Then, combination of genotypes highly ranks those individuals, who have a trait expressed. Usually, the genotypes are scored by the evidence of a genotype–phenotype relationship published in scientific studies. The scores can be revised computationally using genotype data of athletes, if available. From the available genotype data of 180 Lithuanian elite athletes we created an endurance-mixed-power performance TGS profile based on known *ACE* rs1799752, *ACTN3* rs1815739, and *AMPD1* rs17602729, and an emerging *MB* rs7293 gene markers. We analysed an ability of this TGS profile to stratify athletes according to the sport category that they practice. Logistic regression classifiers were trained to compute the genotype scores that represented the endurance versus power traits in the group of analysed athletes more accurately. We observed differences in TGS distributions in female and male group of athletes. The genotypes with possibly different effects on the athletic performance traits in females and males were described. Our data-driven analysis and TGS modelling tools are freely available to practitioners.

## 1. Introduction

Elite athletic status is a polygenic trait with multiple candidate gene variants playing a certain role, either alone or through complex, gene–gene and gene–environment interactions. With the rapid development of molecular research in sport, multiple genetic markers associated with endurance and power physical performance have been discovered [1,2,3]. The meta-analyses of genetic associations with the power or endurance athletic status make these associations more accurate and account for the differential effects between subgroups of sex and race. The most studied polymorphisms *ACE*(rs1799752) and *ACTN3* (rs1815739) have been associated with both: power and endurance athletic performance in multiple studies [4,5,6].

A simple additive model, expressing an integrative effect of multiple markers on a trait, is a total genotype score (TGS). It has been introduced by Williams and Folland [7] and is also known as a genetic predisposition score. Using synthetic data of 23 genetic markers of endurance excellence aggregated into the TGS score they calculated “that there is just a 0.0005% (1 in 200,000) probability that even a single individual exists in the world possessing the optimal DNA variant for endurance performance of all 23 polymorphisms” [7]. Since then, the TGS has been a subject of numerous studies used to model and predict the integrative effect of the DNA markers on the athletic traits [8,9,10,11,12].

### 1.1. Polygenic Profile Expressed through TGS and Trait Predisposition

For the TGS model of the polygenic profile to be useful in practice in predicting a trait, it should correlate with the trait and it has to be accurate in its predictions [13]. In most cases, polygenic profiles are derived from the associations of genetic variants and traits published in scientific literature such as in Jones, 2016 [5] by companies, individuals, or sports geneticists. A certain degree of arbitrariness exists in assigning a score to a genotype in a polygenic model that expresses how favourable for the trait that genotype is. A hypothetical example of one possible assignment is illustrated in Table 1 for a tendon injury profile using data from a published scientific study [14].

In Table 1 for each gene marker there could be also a confidence score assigned by an expert who created that model reflecting how much evidence and support this association received in the scientific literature. The TGS can be weighted by population genotype frequencies [15,16].

To compute TGS, the homozygous and heterozygous genotypes AA, Aa, and aa are assigned numerical scores that express a level of genetic predisposition to the trait of a person who has that genotype. In the original work on TGS of Williams and Folland [7] the TGS score included 23 markers and varied from 0 to 100. However, the range of TGS variation can be set differently in such a way that minimum and maximum values of the TGS score faithfully represent trait extremes. For example, if we were to describe by the TGS a risk of injury, then for those with high injury risk according to their genotypes, the TGS would attain a maximum of 1. For those with non-injury genotypes it would attain a minimum of −1. The athletes with high predisposition to injury based on their genetic profile would have the TGS close to 1 and in no predisposition to risk it would be close to −1. Such models can also be applied in describing the multimarker traits in general population.

Similarly, in the power–endurance axis of athletic performance the TGS may vary from −1 (power only) to 1 (pure endurance) with 0 in between meaning mixed. For the TGS modelled in this way the TGS of pure endurance polygenic profile in endurance athletes would have high positive TGS and in power athletes would have high negative TGS. In athletes practising mixed (power–endurance) sports the expected TGS value would be close to zero.

### 1.2. Practical Application of TGS and Polygenic Profiles

Effects of genetic variants on different athletic traits in different populations and different sports vary greatly. Usually, literature derived models are validated and adjusted by local laboratories and centers using available DNA samples of the athletes. Once the athlete group is genotyped, then the relationships between the TGS and athletic traits can be tested quantitatively in several ways.

For continuous traits a correlation analysis can be performed to measure degree of associations. If the trait is categorical variable, such as endurance, mixed or power athletic status, then analysis of variance (ANOVA) can be performed comparing mean values of TGS to test whether the differences in means of TGS across different athletic status under the study are statistically significant [15]. The TGS can be grouped into levels and converted into the factor. In this case a $\chi^{2}$ or an exact Fisher test can be applied to test whether there is a statistically significant dependency between the TGS levels and the trait. When ANOVA assumptions are violated, the TGS values of athletes from different sport groups can be compared by a Wilcoxon signed rank test. A Kolmogorov-Smirnov test can be applied to compare distributions of the TGS scores either among groups or with the expected distribution.

### 1.3. Lack of Associations

Generally, associations between genetic variants and traits are established by individual laboratories from collected case-control samples by genotyping or in genome wide association studies (GWAS) [17]. Very large samples are required in GWAS. It is very well known that in sports genetics the sample sizes are small and rarely reach over 200 (as an example see Table 1 in Weyerstras, 2018 [18]). For this reason laboratories may not be able to reproduce results reported by others. A varying degree of allele associations with endurance trait is well demonstrated across different populations in meta analysis of genetic polymorphisms in *ACE* and *ACTN3* genes [4]. Varying association levels with physical performance traits can also be seen in other recent meta analysis studies of various markers [19,20,21]. In a study of TGS application to predict power athletic performance in Japanese athletes [22,23] no association was found. Another study showed that TGS cannot be used to identify talent in soccer, but it can be used to choose training method to develop power-based qualities of soccer players [24].

### 1.4. Multimarker Representation

In the presented study, we focused on genetic variants associated with physical performance traits important for elite athlete qualification. The studied polygenic profile consisted of gene markers genotypes: *ACTN3* rs1815739 (RR, RX, XX), *MB* rs7293 (AA, AG, GG), *AMPD1* rs17602729 (CC, CT, TT), and *ACE* rs1799752 (DD, ID, DD).

In the present study, we focused on variants putatively associated with sports performance traits important for elite athlete performance. Inconsistent results have been published regarding the association of *ACE*, *ACTN3*, *AMPD1* genotype with athletic phenotypes. To date, only the *ACTN3* and *ACE* polymorphisms have been associated with either endurance, strength, or power athletic level performance, while the *AMPD1* and *MB* are other candidates providing less consistent results. It has already been shown that many variants which have a significant association with physical performance in several studies of one population may not necessarily have the same association in another.

The choice of these markers for this study was motivated by: (i) the fact that *ACE* and *ACTN3* gene markers are associated with both endurance and power; (ii) the *ACE*, *ACTN3* and *AMPD1* were already used in TGS modelling of the endurance athletic profile [15]; (iii) all four markers were continuously investigated in a cohort of Lithuanian elite athletes [25,26,27].

A brief description of these gene markers in the context of human physical performance are provided in the following.

Genes encoding the angiotensin-converting enzyme (*ACE*) and the α-actinin-3 (*ACTN3*) are two of the most studied “performance genes” and both have been associated with endurance, sprinting, as well as other power phenotypes and elite performance. Circulating *ACE* exerts a tonic regulatory function in circulatory homeostasis. A polymorphism (rs1799752) of the human *ACE* gene (17q22-q24) has been identified in which the presence (insertion, I allele) rather than the absence (deletion, D allele) of a 287-bp Alu-sequence insertion fragment is associated with lower serum and tissue ACE activity. An excess of the I allele has been associated with some aspects of endurance performance. Similarly, several studies have shown the *ACE* D allele to be associated with greater strength and muscle volumes at baseline and an increased percentage of fast-twitch muscle fibres. In addition, the *ACE* D allele was associated with elite power athlete status [25,26].

The human *ACTN3* gene (11q13-q14) encodes the protein α-actinin-3, a component of the contractile apparatus in fast twitch skeletal muscle fibres. A common genetic variation (rs1815739) in the *ACTN3* gene results in the replacement of an arginine (R) with a stop codon (X) at amino acid 577 (p.R577X; C→T transition at position 1747 in exon 16). Homozygosity for the nonsense mutation (XX) within *ACTN3* results in deficiency of α-actinin-3, however it does not result in an abnormal muscular phenotype. It has also been suggested that the X allele may confer an advantage during endurance events. Several case-control studies reported that *ACTN3* RR genotype is over represented or *ACTN3* XX genotype is under represented in power oriented (including sprinting) athletes in comparison with controls. The hypothesis that *ACTN3* R allele may confer some advantage in power performance events was supported by several cross-sectional studies in non-athletes including mouse models of the *ACTN3* deficiency [25,26].

Associations between the *ACTN3* (R577X) polymorphism and power status are inconsistent. The meta-analysis study presented clear associations between the R allele (RR and RX genotype) in the *ACTN3* polymorphism and elite power athlete status. They found significant effects of the R allele overall and in Western and female subgroups, and RX genotype in Asians, and males due to outlier treatment. Interaction analysis improved the outcome in a female subgroup [19].

Adenosine monophosphate deaminase (AMPD1) is a very important regulator of muscle energy metabolism during exercise. The AMPD1, also known as myoadenylate deaminase, predominates in all skeletal muscle fibres. The gene encoding this skeletal muscle-specific isoform (*AMPD1*) is located on chromosome 1 (1p13). *AMPD1* is mainly expressed in fast-twitch (type II) muscle fibres. Differential *AMPD1* gene expression may contribute to quantitative variations in enzyme activity across muscle groups with different types of fibres. The nonsense mutation c.34C>T (C to T transition in nucleotide 34, p.Gln12X, rs17602729) in exon 2 of the *AMPD1* gene converts glutamine codon (CAA) into the premature stop codon (TAA), which results in the early interruption of protein synthesis and appears to be the main cause of AMPD deficiency. This polymorphism (rs17602729) in the *AMPD1* gene is a common polymorphism among Caucasians that can impair exercise capacity. The *AMPD1* C allele may help athletes to attain elite status in power-oriented sports [15,27].

We also included the myoglobin gene given its crucial role on muscle function. Myoglobin (encoding *MB* gene) is a cytoplasmic hemoprotein that is expressed primarily in oxidative skeletal muscle fibres and cardiomyocytes. Myoglobin plays two roles: intracellular oxygen transportation and formation of intracellular oxygen reserve. A polymorphism (rs7293, c.174G>A) in exon 2 of the *MB* gene might be associated with physical performance in elite athletes [28].

### 1.5. Differences between Genders

The female and male athletes are different phenotypically. They also may differ in distributions of genotypes of gene markers of physical performance. For example, gender effects with respect to *ACTN3* were noted in a study of athletes of six Balkan populations [29]. The XX genotype was twice prevalent in males than females (15.7% vs. 7.4%). The RR genotype was the most abundant in females, while the heterozygous genotype was prevalent in males in jointly analysing all tested individuals. In that study, gender seemed to have the strongest influence on the overall results in Albanian population and FYR Macedonia. Only one XX genotype was present in one female from Albania. It was not detected in Albanian females from FYR Macedonia (see Table 1 in Konakli. 2017) [29].

### 1.6. Contribution of Our Study

In this study, we report observed differences between polygenic profiles of genders expressed by TGS in Lithuanian athletes practising endurance, power, and mixed sports. In the analysed group of athletes the females and males differ in allele composition favouring achievement of high endurance or power performance. The four gene markers *ACTN3* rs1815739, *MB* rs7293, *AMPD1* rs17602729, and *ACE* rs1799752 in the group of 180 Lithuanian elite athletes were already extensively analysed in other case-control association studies [25,26,27]. In this study we focus on the total genotype score of these markers that we believed to be important for explaining individual variations in sports performance.

## 2. Materials and Methods

### 2.1. Subjects and Ethics Approval

All procedures in this study conformed with the ethical standards in Sport and Exercise Science Research and were approved by the Lithuanian Bioethics Committee. Written informed consent was obtained from all participants and the study was conducted in compliance with the Declaration of Helsinki. The study involved 180 Lithuanian elite athletes (130 males and 50 females, participants and winners in major international competitions, including European Championships, World Championships and Olympic Games; aged 26.4 ± 6.7 years). The athletes were stratified into three groups according to the duration and distance of the event, in sports disciplines that ranged from endurance-oriented to power-oriented sports. The endurance group (*n* = 81) included very long (race duration ≥ 30 min), long (race duration 5–30 min), and medium (race duration 45 s to 5 min) distance athletes: skiers, road cyclists, bi-athletes, long-distance runners, modern pentathletes, swimmers, and rowers. The sprint/power group (*n* = 44) included sprinters and other power athletes with predominantly anaerobic energy production: sprinters, jumpers, and throwers. The mixed group (*n* = 55) comprised athletes whose sports utilised mixed anaerobic and aerobic energy production (team sports): tennis players, handball players, and footballers.

### 2.2. Genotyping

Genomic DNA was extracted from peripheral blood leukocytes by the standard phenol-chloroform extraction method. Genotyping of the gene polymorphisms was performed using polymerase chain reaction (PCR). PCR was used to detect the I and D alleles of the *ACE*(rs1799752) gene according to the method described by Gineviciene et al. [25,26], using upstream primer 5${}^{\prime }$-CTGGAGACCACTCCCATCCTTTCT-3${}^{\prime }$ and the downstream primer 5${}^{\prime }$-GATGTGGCCATCACATTCGTCAGAT-3${}^{\prime }$. This method yields PCR fragments of 190 bp and 477 bp in the presence of the D and the I alleles, respectively. Reaction products were visualised by electrophoresis on a 2% agarose gel and identified by ethidium bromide staining. The resulting PCR products of *AMPD1*(rs17602729), *ACTN3*(rs1815739), and *MB*(rs7293) were genotyped by restriction fragment length polymorphism analysis [25,26,27,30]. The primer sequences were as follows: *AMPD1* (rs17602729) forward primer 5${}^{\prime }$-CTTCATACAGCTGAAGAGACA-3${}^{\prime }$ and reverse primer 5${}^{\prime }$-GAATCCAGAAAAGCCATGAGC-3${}^{\prime }$; *ACTN3* (rs1815739) forward 5${}^{\prime }$-CTGTTGCCTGTGGTAAGTGGG -3${}^{\prime }$ and reverse 5${}^{\prime }$-TGGTCACAGTATGCAGGAGGG -3${}^{\prime }$ primers; *MB* (rs7293) forward primer 5${}^{\prime }$-TGAAGTCAGAGGACGAGATGAATGC-3${}^{\prime }$ and reverse primer 5${}^{\prime }$-GCCCAGGCTCTGCCTCCTACCTCCAG-3${}^{\prime }$. The amplified fragment subsequently underwent digestion by NspI endonuclease for *AMPD1* (rs17602729), DdeI for *ACTN3*(rs1815739), and BsaHI for *MB*(rs7293) (Thermo Fisher Scientific, Lithuania). Digested PCR fragments were separated by 2% agarose gel electrophoresis, stained with ethidium bromide, and viewed in UV light.

### 2.3. Computation of TGS Scores of Multimarker Profiles of Athletes

The genotypes of *n* gene markers comprise individual genetic profiles. For the *i*th marker having genotypes AA, Aa, and aa, lets denote its genotype scores by $G_{i}$. In the original work of Williams and Folland [7] $G_{i}\in \left(2,1,0\right)$ corresponding to favourable, medium, and neutral genetic predisposition towards a trait: $G_{i}\left(aa\right)=2$, $G_{i}\left(Aa\right)=1$ and $G_{i}\left(AA\right)=0$. The TGS of the individual profile of *n* gene markers is defined as a normalised sum of genotype scores $TGS=\left(100/2n\right)\times \left(G_{1}+\ldots +G_{n}\right)$.

Gene markers may have different strength of association with the traits of interest which can be modelled numerically by weighting. Let us denote by $g^{i}$ a magnitude of association between a genotype *i* of gene marker *m* and a trait *t*. Then, the weighted TGS for the trait *t* is defined as:

(1) $$TGS_{t}=\sum_{m}^{M}G_{m}^{i}g^{i},\;G_{m}^{i}\in \left(-1,0,1\right),\;g^{i}\in \left[0,1\right],\;m=\left(1,..,M\right).$$

where *i* denotes a gene marker genotype, *M* is a number of gene markers *m* in a profile and $G_{m}^{i}$ is a score which attains following values: $G_{m}^{i}=1$ for a genotype that strongly predisposes to the trait *t*; $G_{m}^{i}=0$ for neutral or midway genotype and $G_{m}^{i}=-1$ for genotype predisposing to the opposite direction of the trait. The genotype weights $g^{i}$ can be assigned from the evidence in literature or derived computationally. Since $G_{m}^{i}$ in this formulation expresses a direction of association, the weights $g^{i}$ in the TGS formulation in Equation (1) determine the genotype scores.

Numbers of gene markers that are associated with traits may vary. This variability may impair a comparison of the TGS between the different traits and different sets of markers of the same trait. The TGS should be uniform across the traits and profiles in reporting to which end of a trait an individual genetic profile places its owner. Therefore, values from the interval $\left[TGS_{min},TGS_{max}\right]$ are normalised by mapping this interval linearly to the [−1,1] interval.

In this study we will highlight two TGS models. A model based on the original work of Williams and Folland [7] called WF TGS and a model in which the genotype scores were derived from data using logistic regression classifier called LR TGS.

### 2.4. Computational Tools

We wrote Python scripts to model a polygenic profile and to compute its TGS scores. Application of these tools on Lithuanian athlete data and all analysis and computations performed in this study are reproducible and documented in a publicly available Python notebook in GithHub (see data availability). We used *pandas* [31] and *numpy* [32] libraries for data processing. The *scipy* [33] library functions were used to compute $\chi^{2}$ and Wilcoxon rank sum tests where appropriate. The logistic regression (LR) classifier from *scikit-learn* [34] linear model library was used to derive weights and genotypic scores computationally from the athletes data. The *seaborn* [35] and *matplotlib* [36] libraries were used to visualise the results.

### 2.5. Logistic Regression Classifier to Derive Genotype Scores

Logistic regression (LR) classifier is a binary classifier that classifies *n*-dimensional feature vectors $\left(x_{1},x_{2},\ldots ,x_{n}\right)$ into two classes c=0 or c=1 by computing a probability of a class *c* of a feature vector as [34]:

(2) $$p\left(x_{1},x_{2},\ldots ,x_{n}\right)=\frac{1}{1+exp(-\left(b_{0}+b_{1}\times x_{1}+\ldots +b_{n}\times x_{n}\right))}\;p\left(x_{1},x_{2},\ldots ,x_{n}\right)\in \left[0,1\right].$$

The LR coefficients $b_{0},b_{1},\ldots ,b_{n}$ are estimated in classifier training. Training data for the classifier are feature vectors representing genotypes of the four gene markers of athletes from the three sports classes—endurance, mixed, and power. For example, a feature vector and its values can be as follows $x=\left(x_{1},x_{2},x_{3},x_{4}\right)=\left(DD,RX,AG,CT\right)$. To train a logistic regression classifier the categorical features should be transformed into numerical values by creating dummy variables. The encoding is also known as one-hot encoding in machine learning. In on-hot encoding a 4-dimensional feature vector would be transformed into a 12-dimensional vector in which each level of the categorical variable is represented by a new dimension: $\left(x_{1}^{DD},x_{1}^{DI},x_{1}^{II},x_{2}^{RR},x_{2}^{RX},x_{2}^{XX},x_{3}^{AA},x_{3}^{AG},x_{3}^{GG},x_{4}^{CC},x_{4}^{CT},x_{4}^{TT}\right)=\left(1,0,0,0,1,0,0,1,0,0,1,0\right)$. After the transformation each feature vector of a polygenic profile of an athlete becomes a binary feature vector indicating a presence or absence of a specific genotype in the profile.

For binary features $x_{i}=\left(1,0\right)$ computation of class probability in the logistic regression depends only on the feature weights $b_{i}$. Due to the nature of the feature vectors, the coefficients $b_{i}$ represent both: a magnitude and a direction of the effect of the genotype on the class probability.

For example, let us suppose that endurance class has label 1 and power class has label 0. Let us assume that a trained LR classifier has following weights: $b_{0}=0$, $b_{DD}=0.4$, $b_{RX}=-0.07$, $b_{AG}=0.24$, and $b_{CT}=0.1$. For a feature vector x=(DD,RX,AG,CT) only weights of the respective genotypes matter in linear combination $y=b_{0}+b_{DD}\times x_{1}^{DD}+\ldots +b_{CT}\times x_{4}^{CT}$ yielding y=0.4+(−0.07)+0.24+0.1=0.65. By inserting *y* into p(y)=1/(1+exp(−y)) gives 0.657 which is a class probability of the feature vector x=(DD,RX,AG,CT). By its magnitude the class probability assigns feature vector *g* to the endurance class (p(y)>0.5).

In two class classification where the classes represent two opposite expressions of a trait, coefficients $b_{i}$ can be used as genotype scores in polygenic model to compute TGS. Positive coefficients will sum up in favour of the class 1—and negative coefficients—in favour of class 0.

### 2.6. Determining Genotype Scores from the Coefficients of a Fitted LR Classifier

In this study, we derived genotype scores from the coefficients of a logistic regression (LR) classifier fitted in two class classifications: endurance (class 1) versus power (class 0); endurance (class 1) versus mixed (class 0) and power (class 1) versus mixed (class 0). Classification accuracy was estimated in a 10-fold cross-validation. Final LR classifiers were trained on all available data. The coefficients at each genotype feature and class probabilities are shown in Table 2. Since the Table 2 is instrumental in computing the genotype scores it is placed in Methods section.

A process of inference of the genotype scores for the TGS model can be thought of as a classifier combination scheme in which a pattern is assigned to a class, that is supported by a majority in the classifier committee. The signs of the genotype scores computed from the coefficients *b* determine the direction towards the trait extremes: endurance 1 and power −1; a mixed class is assumed to be a midway 0.

For example, let us analyse inference of genotype scores of *ACE* genotype DD in Table 2. In endurance versus power (class 1 vs. 0) and in endurance versus mixed (class 1 vs. 0) classification the respective class probabilities are 0.602 and 0.603 in favour of the endurance class. The magnitudes of coefficients $b_{EvsP}=0.415$ and $b_{EvsM}=0.418$ are averaged to determine a magnitude of the *ACE* DD genotype score of 0.4165.

For *ACE* genotype II in E(1) vs P(0) classification the class probability is 0.351 in favour of the power class; in the E(1) vs M(0) it is 0.407 in favour of the mixed class and in P(1) vs M(0) it is 0.579 in favour of the power class. Two classifiers supported the power class. Therefore, the direction of the *ACE* II genotype score should be negative towards the power class. The magnitude of the *ACE* II genotype score can be computed as an average of the two absolute values |−0.610| and |0.321| resulting in the *ACE* II genotype score of −0.4655. For the mixed class the genotype scores are set close to 0 because of how the TGS model is constructed. For example, the *AMPD1* CT genotype strongly supports mixed class and its genotype score in the TGS model will be set to 0. The inferred genotype scores and their supported traits are presented in the results sections.

### 2.7. Probabilities of Genotype Combinations in Population

Frequency of *i*th genotype $gt_{m}^{i},i\in \left(aa,aA,AA\right)$ of some gene marker *m* in population usually can be found in public population databases, such as Ensembl [37] or GnomAD [38], or estimated from a control dataset. These frequencies can be assumed as empirically estimated genotype probabilities $p(gt_{m}^{i})$ in a population. Assuming that gene markers m=(1,…,M) are independent of each other, one can compute a probability of a specific genotype combination as $p\left(gt_{m_{1}}^{i},\ldots ,gt_{m_{M}}^{i}\right)=p\left(gt_{m_{1}}^{i}\right)\times \ldots \times p\left(gt_{m_{M}}^{i}\right)$, where *i* can be only one of the three possible genotypes (aa,aA,AA). Let us denote this probability of a genotype combination of the *M* gene markers as $p\left(M\right)=p\left(gt_{m_{1}}^{i},\ldots ,gt_{m_{M}}^{i}\right)$.

We can perform a single experiment by selecting one individual from a population carrying a specific combination of genotypes. This experiment has only two outcomes—a success: the individual has the specific genotype combination; and a failure: he has not. The probability of success in this experiment is p(M).

We can independently select *n* individuals from a population. Each time there will be only two outcomes: success or failure. Out of *n* independently selected individuals (experiments) there might be a number of individuals *k* who will carry that specific combination of genotypes. The probability that *k* out of *n* independently selected individuals will carry the specific genotype combination—*k* successes in *n* trials with a probability of success p=p(M)—is given by a binomial distribution which is a discrete probability distribution:

(3) $$P_{p}\left(k|n\right)=\frac{n!}{(n-k)!k!}\times p^{k}\times {\left(1-p\right)}^{n-k}.$$

In Equation (3), the $P_{p}\left(k|n\right)$ is a probability to observe *k* individuals out of *n* randomly selected individuals from a population carrying a specific genotype combination, the probability of which is p=p(M). Using this scheme we can explore which and how many genotype combinations are over-represented in the group of athletes compared to a general population. We know how many athletes *k* out of n=180 have a specific genotype combination. Therefore, we can compute how likely it is to encounter the same number *k* of individuals in a randomly selected 180 group from a general population carrying the same genotype combination as in athletes.

## 3. Results

### 3.1. Genotype and Allele Frequencies

The genotype and allele counts in Lithuanian elite athletes and controls are presented in Table 3.

Differences in genotype frequencies between athletes and controls are observed for ACE and MB gene markers. Differences in allele distribution for ACE and ACTN3 gene markers. In the current study, a distribution of genotypes of AMPD1 gene marker did not show significant differences between the athletes and controls as in previous study [27]. Here we studied a smaller group of athletes who were matched by genotyping all four markers.

### 3.2. Multimarker Genotype Combinations in the Group of Lithuanian Athletes

**Figure 1:**
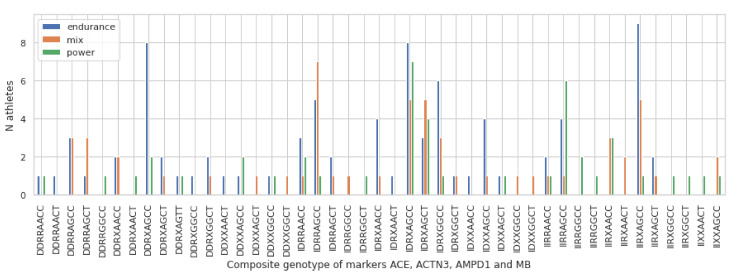
Representation of a number of athletes in endurance, mixed, and power sports carrying a specific genotype combination.

In the group of the analysed Lithuanian athletes out of the 81 possible unique genotype combinations (3 genotypes of 4 genes $3^{4}$) there were 45 combinations of the genotypes of four gene markers *ACE* rs1799752 (DD, ID, II), *ACTN3* rs1815739 (RR, RX, XX), *MB* rs7293 (AA, AG, GG), and *AMPD1* rs17602729 (CC, CT, TT). Counts of genotype combinations in athletes of different sports are shown in Figure 1.

Some genotype combinations are more frequent among athletes of all three sport groups: 20 athletes have the ID-RX-AG-CC and 12 have ID-RX-AG-CT genotype combinations. Other, more frequent genotype combinations occur in endurance and mix sport groups: 15 athletes have II-RX-AG-CC and 10 have DD-RX-AG-CC genotype combinations. In the power and mixed sports group 11 athletes have II-RR-AG-CC genotype combination. Ensembl database shows that world-wide population frequencies of the genotypes for all markers are quite common, as well as in Lithuanian controls.

Table 4 shows genotype combinations ordered by the ascending binomial probability of how likely it is to see a specific genotype combination as many times as in athletes in a group of 180 randomly selected individuals from a general Lithuanian population. The probability of success p(M) for each combination of genotypes was computed using genotype frequencies in Lithuanian controls (Table 5). Data are shown only for $P_{p(M)}\left(k|180\right)<0.13$.

The results of this calculation suggest that most frequent genotype combinations observed in athletes group might be less frequent in general Lithuanian population and more specific to the athletes.

### 3.3. Genotype Scores in the Tgs for Endurance and Power Performance Traits in Athletes

In a traditional approach to TGS computation, first introduced by Williams and Folland (WF), genotypes reflecting a favourable, a medium, and a neutral effect of a genotype on a trait have respective values 2, 1, 0 that are based on the evidence in scientific literature. In this study, the genotype scores in WF TGS model are based on the previously published data [25,26,27,30]. Table 5 shows genotype scores and associated traits for the WF TGS model and also for the LR TGS model in which genotype scores were derived from the coefficients of a fitted logistic regression (LR) classifier.

To derive genotype scores (Table 5) in the LR TGS model we trained three logistic regression classifiers using all athletes genotype data: endurance versus power, endurance versus mix and power versus mix. Obtained coefficients of the LR classifiers, shown in Methods (Table 2), were used to compute genotype scores in Table 5, as explained in Methods.

10-fold cross validation classification accuracy and confusion tables of final classifiers are shown in Table 6. In all cases, the classification accuracy in 10-fold cross validation is very poor. However, from confusion tables it is seen that athletes in endurance class are classified correctly as endurance much better compared to the power and mix classes: 92% in endurance versus power classification and 85% in endurance versus mix classification. Additionally, athletes in mix class are classified correctly as mix 78% in power versus mix classification.

Features used in classification were only genotypes of four gene markers and sample size was rather small in these classification tasks. Still, the results show that endurance athletes have a signature of genotype combinations that allows to discriminate them from other classes, regardless of poor overall classification accuracy.

We further investigated whether the genotype scores computed from the coefficients of the trained logistic regression classifier and used in the TGS model lead to a better characterisation of athlete’s endurance or power traits by the TGS. In our study we called this model the LR TGS model. As a reference point for comparison we used the TGS computed with genotype scores of (2, 1, 0) linearly mapped to the interval [−1,1]. We called this model WF TGS model.

### 3.4. TGS Based on Genotype Scores Derived from the Coefficients of LR Classifiers

**Figure 2:**
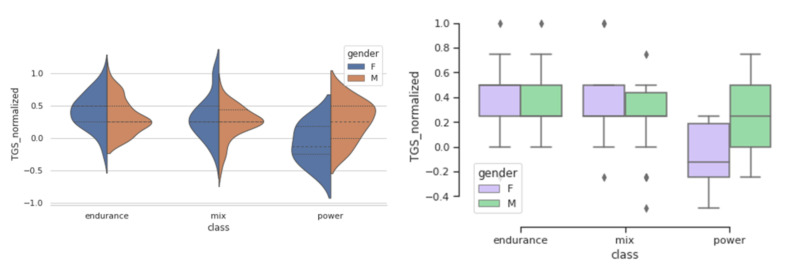
WF TGS value distributions shown by violin and box-whisker plots in females and males in different sport categories.

**Figure 3:**
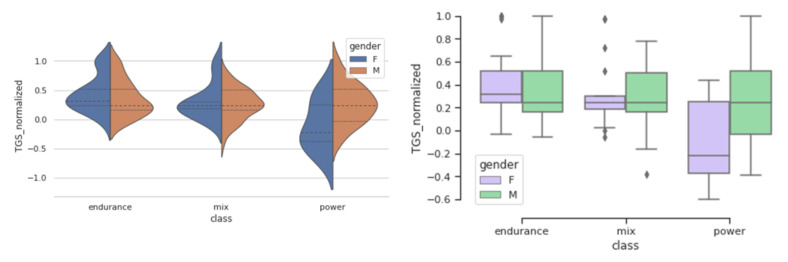
LR TGS value distributions shown by violin and box-whisker plots in females and males in different sport categories. Violin plots show areas of high data point density along the axis of value distribution.

TGS is an integrative metric, representing the additive effect of all genotypes in predicting a trait. We analysed how LR TGS and WF TGS values are distributed in endurance, mix, and power groups. Figure 2 and Figure 3 represent distribution of LR TGS and WF TGS for genders in different sport groups and show how these TGS distributions differ between endurance, mix, and power sports, and also between males and females.

A significance of these differences was tested by a Wilcoxon signed rank test which was chosen because of small sample sizes and TGS normality assumption may not hold. The results of the test are summarised in Table 7.

A number of females in the power class is very small, therefore, the obtained results are only suggestive. However, in the comparison of the endurance versus power groups a more significant difference is detected using the LR TGS values, meaning that the LR TGS is more representative of differences between these groups.

**Figure 4:**
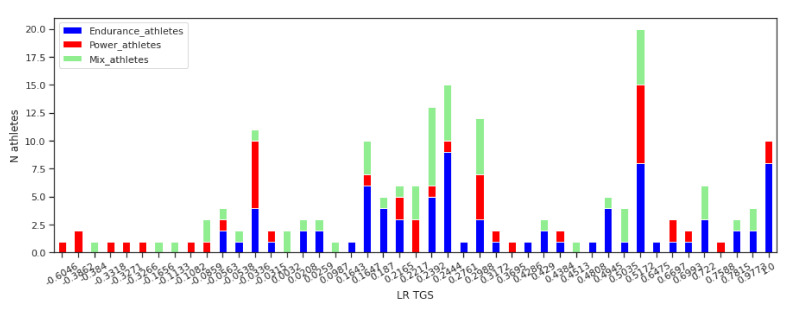
Athletes data order by the LR TGS values. The graph shows a number of athletes from the different sports characterised by the same LR TGS value. The order is from the negative TGS values representing the power class to positive, representing the endurance class.

**Figure 5:**
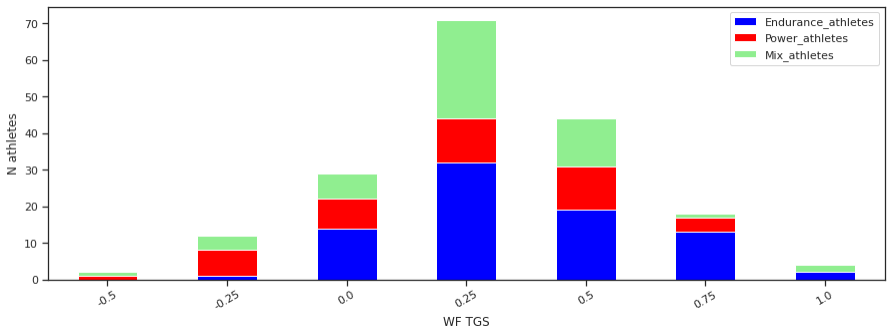
Athletes data order by the WG TGS values. The graph shows a number of athletes from the different sports characterised by the same WF TGS value. Values are ordered from negative representing the power class towards positive, representing the endurance class.

The TGS orders genotype combinations across the endurance, mixed, and power athletic performance axis. Figure 4 shows ordering of the athletes in the respective sport groups by the LR TGS values. In Figure 5 this ordering is shown for the WF TGS values. The graphs show a number of athletes from the different sports characterised by the same LR TGS value. The athletes are ordered according the TGS value from the negative TGS values representing the power class towards positive, representing the endurance class.

Figure 4 and Figure 5 clearly show the difference between the LR TGS and WF TGS. The former is more granular and represents a variability in genotype combinations better. Although the latter gets concentrated in a small number of levels. In both cases, the athletes from the power group are represented by the range of the negative TGS values (normalised in both cases). However, we observe that there is a significant overlap between the three sport classes in genotype combinations and their corresponding TGS values. A majority of the TGS values are not pure power or endurance or mix.

In Figure 4 there are instances of the athletes from the power class to be placed at the positive endurance end and vice versa. Similarly, the positive WF TGS values also represent both: endurance at a higher extent and power at a lesser extent. This means that the some genotypes have influence in both directions—power and endurance.

### 3.5. Differences between Females and Males

In this study, we observe differing distributions of the TGS values in female and male groups (Figure 2 and Figure 3). This may happen if the individual genotypes have different influence of the trait in females compared to males. Table 8 shows distribution of athletes by genotype, by gender and by the sport category.

The good classification of the sport groups by the genotypes depends on the prevalence of a discriminating genotype in one group and a lack of it in the other group. Analyzing prevalences of the the particular genotypes in the three sports groups in female and male athletes (comparing percentages of individual genotypes in any sport group between females and males) showed several genotypes that may have opposite effects on the endurance or power performance trait in males and females.

In our data, several genotypes can be identified as acting on opposite sides in females and males. The *AMPD1* rs17602729 TT genotype is present in *power females*, but in *endurance males*. However, there are only two athletes with this genotype. The *MB* rs7293 genotype GG prevails in *power females* (50%) but in *endurance males* (42%); the genotype AA prevails in *endurance females* (62%) but in *power males* (41%). The *ACTN3* rs1815739 XX is not present in power females, but it is present in both endurance and power males. The observed imbalances are more characteristic to the genotypes that have lesser frequency among analysed athletes and also in the general population (*AMPD1* TT and *ACTN3* XX genotypes).

### 3.6. Sample Size Considerations

In sports genetics the sample sizes are naturally limited, especially in small populations since they do not have many elite athletes. For groups in Table 7 an actual effect size and an empirical power (Table 9) of Wilcoxon test was computed by using G*power [39]. The power is a complement of the type II error which is a probability to falsely reject the null hypothesis of no difference.

The large effect sizes (above 1) show that the actual sample sizes available for analysis (*n2* = 6, *n1* = 38, 27, 17) are sufficient to demonstrate differences between the elite female athletes in power sports versus males and females in endurance and mixed sport groups by the total genotype score. Same holds for the athletes in the power and endurance groups. We detect large effect size which does not require a large sample size, however replication of calculations with somewhat bigger groups might be advisable. The smallest observed effect size is in endurance versus mixed athletes groups, in which the TGS differences are not significant. This is somehow expected since the mixed sports athletes share features of both sports groups: endurance and power. Overall actual sample sizes of elite athletes in this study do not diminish a value of the the total genotype score algorithm to reflect opposite traits of athletic performance and reveal differences between the athletes that otherwise would be difficult to notice.

## 4. Discussion

Multimarker representations of human performance traits are usually derived from published experimental evidence and quite often the reported association vary considerably across populations and sports. We analysed a group of elite Lithuanian athletes aiming to find out whether and how genotype combinations of four genetic markers stratify athletes according to different sport category. This is a novel report using actual data on the polygenic profile of elite athletes with the same ethnic origin.

We created an endurance-mixed-power performance profile from experimental data based on known *ACE* rs1799752, *ACTN3* rs1815739 and *AMPD1* rs17602729, and emerging *MB* rs7293 gene markers. We analysed the total genotype score TGS computed from this profile in the group of Lithuanian athletes and gained several important insights. First, the additive effect of genotypes on the trait can be different in female and male athletes, implying that TGS may have to be constructed differently for each gender. Second, if scores of the genotypes in the TGS model are computed from the original athletes genotype dataset, then the total genotype score TGS stratifies athletes into their respective sport categories more accurately compared to the TGS constructed by the original method of Williams and Folland.

The use of a total genotype score in practice, so far, is limited to the exploratory data analysis [11,12,22,24,40]. Its usage in talent prediction is premature and was deemed ineffective [41]. However, the TGS was found as a good tool to complement analysis of predictive power of individual markers in diabetes study [42].

In our study, we used TGS to characterise Lithuanian elite athletes practising power, mixed, and endurance sports. We constructed and analysed a novel data-driven method to compute the genotype scores in the TGS from the experimental athlete’s data. We trained logistic regression classifiers on the sole genotype data of the athletes and computationally derived genotype scores from the coefficients of the LR classifiers. Although we used the LR classifiers, any classification method can be used for this purpose, as long as it allows to infer a magnitude and a direction of the genotype effect on the trait.

It is known that in different populations and different sports the associations between the gene markers and traits of athletic performance may be different [18]. In these situations, a data-driven modelling of the TGS may produce more accurate results because it captures features of a particular dataset that makes it different from others. In our study, through the data-driven TGS modelling we identified and described differences in genotype effects on the power and endurance traits between male and female athletes, that also were noted by other researchers [29].

The main limitation of our study is the small sample size. Another limitation is an assumption that effects of the genotypes on the athletic power or endurance performance traits are additive and independent of each other which is difficult to test due to the small sample size. However, in many cases the datasets for the athletic performance are small [18] due to the objective fact that elite athletic performance is a rare trait and not much elite athlete’s data are available in various populations. However, this does not diminish the descriptive value of our study.

Together with the known markers (*ACE* rs1799752, *ACTN3* rs1815739, and *AMPD1* rs17602729) we analysed an emerging marker of physical human performance *MB* rs7293. There is not much data in literature about this marker [28]. It was hypothesised that the *MB* AA genotype of this marker is related to improved endurance performance. Our computational analysis suggests that *MB* AA genotype might be related to endurance, and GG genotype to power performance in females, but in opposite relationship with the trait in males—AA genotype related to power, and GG genotype to endurance.

## 5. Conclusions

A most important aspect of a TGS is how the genotypes in the multimarker representation are assigned their scores. The scores should faithfully represent the impact of the genotype on the trait so that genotype combination ranks highly those individuals with particular genotype, who have a trait expressed. Most of the time, the genotypes are scored by genotype–phenotype relationship evidence in published scientific studies. A complementary approach is computation of the genotype scores from available experimental data.

In the presented study, we created an endurance-mixed-power performance profile based on known and emerging gene markers and analysed its ability to stratify Lithuanian elite athletes according to the sport category that they practice. Analysis of the total genotype score computed from this profile in the group of Lithuanian athletes showed differences in TGS distribution in females and males. This indicates that the effect of the same genotype on a trait might be different in female and male athletes and this difference should be taken into account if one uses TGS in some practical applications. We explained in detail a procedure of the data-driven derivation of the genotype scores in the TGS model and contributed Python code of our analysis making it reproducible. 

## Figures and Tables

**Table 1:** An example of possible polygenic profile of tendon injury with scored genotypes and population frequency in Ensembl database.

Gene Marker	Genotype	Genotype Score	Trait Outcome	Population Frequency
*COL5A1*	TT	1	High risk	0.157
rs12722	CT	0.5	Moderate risk	0.384
Confidence 1	CC	−1	Low risk	0.458
*COL1A1*	CC	0.5	Moderate risk	0.833
rs1800012	AC	0.5	Moderate risk	0.151
Confidence 1	AA	−1	Low risk	0.016
*MMP3*	AA	1	Increased risk	0.126
rs679620	AG	0	Moderate risk	0.444
Confidence 0.75	GG	−1	Low risk	0.430

**Table 2:** Coefficients of the logistic regression model fitted to the athletes genotype data in the three two class classification tasks: endurance versus power (E vs. P), endurance versus mixed (E vs. M), power versus mixed (P vs. M). The column b represents a coefficient of a corresponding genotype in a fitted logistic regression model. The class probability represents $p\left(class\right)=\frac{1}{1+exp(-b)}$ which is a measure of how strongly a coefficient *b* alone predicts either of the binary classes.

Classification	E(1) vs. P(0)	Class	E(1) vs. M(0)	Class	P(1) vs. M(0)	Class
Genotype	$b_{\mathrm{EvsP}}$	Prob.	$b_{\mathrm{EvsM}}$	Prob.	$b_{\mathrm{PvsM}}$	Prob.
*ACTN3* RR	−0.080	0.479	−0.199	0.450	0.039	0.509
*ACTN3* RX	0.289	0.571	0.241	0.560	−0.071	0.482
*ACTN3* XX	−0.209	0.447	−0.041	0.489	0.032	0.508
*AMPD1* CC	0.288	0.571	0.222	0.555	−0.047	0.488
*AMPD1* CT	0.062	0.515	−0.453	0.388	−0.403	0.400
*AMPD1* TT	−0.350	0.413	0.230	0.557	0.451	0.610
*MB* AA	0.088	0.522	0.117	0.529	−0.068	0.482
*MB* AG	0.223	0.555	0.095	0.523	−0.125	0.468
*MB* GG	−0.311	0.422	−0.212	0.447	0.194	0.548
*ACE* DD	0.415	0.602	0.418	0.603	−0.071	0.482
*ACE* ID	0.194	0.548	−0.044	0.488	−0.250	0.437
*ACE* II	−0.610	0.351	−0.374	0.407	0.321	0.579

**Table 3:** Genotype and allele counts of gene markers *ACE* rs1799752, *ACTN3* rs181573, *AMPD1* rs17602729, and *MB* rs7293 in Lithuanian elite athletes and controls. The *p*-value of $\chi^{2}$ test result of genotype and allele frequencies between elite athletes and controls ${}^{**}$ significant at a level p≤0.05 and ${}^{*}$ significant at a level p≤0.1.

Gene/Group	Genotype Frequency			*p*-Value ($\chi^{2}$)	Allele Frequency		*p*-Value ($\chi^{2}$)
*ACE*	DD	ID	II	0.049 **	D	I	0.093 *
Athletes	46	84	50		176	184	
Controls	63	94	98		220	290	
*ACTN3*	RR	RX	XX	0.118	R	X	0.0786 *
Athletes	56	102	22		214	146	
Controls	104	125	26		333	177	
*AMPD1*	CC	CT	TT	0.625	C	T	0.539
Athletes	133	45	2		311	49	
Controls	184	65	6		433	77	
*MB*	AA	AG	GG	0.0004 **	A	G	0.588
Athletes	35	116	29		186	174		
Controls	69	116	70		254	256		

**Table 4:** Binomial probability $P_{p(M)}\left(k|180\right)$ to observe k individuals in a random group of n=180 carrying a genotype combination of probability p(M).

Genotype Combination	*k*	p(M)	$P_{p(M)}\left(k|180\right)$
ID-RX-AG-CT	12	0.021	0.000345
ID-RX-AG-CC	20	0.0594	0.002910
II-RX-GG-CC	1	0.0374	0.007327
DD-RX-AG-TT	2	0.0013	0.021598
ID-RR-GG-CC	1	0.0299	0.023502
ID-RR-AG-CC	13	0.0495	0.047800
ID-XX-AG-CC	5	0.0124	0.049164
ID-RX-GG-CC	10	0.0359	0.054216
II-RX-AG-CC	15	0.0618	0.055684
DD-RX-GG-CC	1	0.0240	0.055847
II-RR-GG-CC	2	0.0311	0.056269
DD-RX-AG-CC	10	0.0398	0.076339
DD-RR-GG-CC	1	0.0200	0.096777
DD-RR-AG-CT	4	0.0117	0.099880
II-RR-AG-CC	11	0.0515	0.104875
DD-XX-AG-CC	3	0.0083	0.125010

**Table 5:** Gene markers, their genotypes, genotype scores, and associated traits in LR TGS and WF TGS models, along with the population genotype frequencies in the Ensembl database (ACE rs1799752 frequencies obtained from the rs4341 in 100% LD with I/D polymorphism II = CC, ID = GC, DD = GG) and in Lithuanian controls (LR—logistic regression; TGS—total genotype score; WF—Williams and Folland genotype score; Freq.—genotype frequencies; End—endurance-oriented athletes; Mix—mixed athletes group; Pow—power-oriented athletes).

Gene Marker *m*	Genotype *i*	LR TGS $G_{m}^{i}*g^{i}$ Score	Trait	WF TGS $G_{i}$ Score	Trait	Freq. Ensembl All	Freq. Control *n* = 255
*ACE*	DD	0.4165	End	2	End	0.237	0.247
rs1799752	ID	−0.1470	Mix	1	Mix	0.466	0.369
	II	−0.4655	Pow	0	Pow	0.297	0.384
*ACTN3*	RR	−0.0595	Pow	2	End	0.382	0.408
rs1815739	RX	0.265	End	1	Mix	0.435	0.49
	XX	−0.1205	Pow	0	Pow	0.183	0.102
*AMPD1*	CC	0.255	End	2	End	0.927	0.722
rs17602729	CT	0	Mix	1	Mix	0.07	0.255
	TT	−0.4005	Pow	0	Pow	0.003	0.024
*MB*	AA	0.1325	End	2	End	0.313	0.271
rs7293	AG	0.159	End	1	Mix	0.437	0.455
	GG	−0.2525	Pow	0	Pow	0.251	0.275

**Table 6:** Classification accuracy of logistic regression classifiers in three two-class classification tasks. Accuracy is presented as mean ± standard deviation. Confusion tables show how many athletes were classified correctly and incorrectly by final LR classifiers trained on all data.

Classifier	Accuracy m±sd	Confusion Table			
Endurance vs. Power	0.663±0.086			Predicted	
				Pow	End
		Actual	Pow	14	30
			End	6	75
Endurance vs. Mixed	0.522±0.092			Predicted	
				Mix	End
		Actual	Mix	10	45
			End	12	69
Power vs. Mixed	0.476±0.142			Predicted	
				Mix	Pow
		Actual	Mix	43	12
			Pow	24	20

**Table 7:** Statistically significant Wilcoxon rank sum test outcomes (*p*-values at 95% and 90% levels of significance) of LR TGS and WF TGS value comparisons between males and females and different sport groups.

Groups Compared (n)	LR TGS (*p*-Value)	WF TGS (*p*-Value)
Endurance (81) vs. power (44)	0.0396	0.0528
Endurance (81) vs. mixed (55)	0.0861	0.105
Power females (6) vs. males (38)	0.042	0.0229
Females endurance (27) vs. power (6)	0.0152	0.0033
Females power (6) vs. mixed (17)	0.08	0.0022

**Table 8:** Distribution of athletes by genotype, by gender, and by sport category.

Gene Marker	Genotype	Females *n* = 50			Males *n* = 130		
		Endurance	Power	Mixed	Endurance	Power	Mixed
		*n* = 27	*n* = 6	*n* = 17	*n* = 54	*n* = 38	*n* = 38
*ACE*	DD	10	2	4	15	7	8
rs1799752	ID	15	1	7	24	16	21
	II	2	3	6	15	15	9
*ACTN3*	RR	9	4	3	13	12	15
rs1815793	RX	15	2	12	35	20	18
	XX	3	0	2	6	6	5
*AMPD1*	CC	20	3	11	43	30	26
rs17602729	CT	7	2	6	10	18	12
	TT	0	1	0	1	0	0
*MB*	AA	8	0	5	8	9	5
rs7293	AG	16	1	10	38	25	26
	GG	3	5	2	8	4	7

**Table 9:** Actual effect size and empirical power of the statistical Wilcoxon test with the available sample sizes (*n1* and *n2*) for the LR TGS model of the groups shown in Table 7.

Groups	Sample Size	Actual	Actual	Empirical
	*n1, n2*	Effect Size	α	Power
All athletes endurance vs. power	81, 44	0.55	0.05	0.81
All athletes endurance vs. mixed	81, 55	0.379	0.1	0.68
Power males vs. females	38, 6	1.057	0.1	0.75
Females endurance vs. power	27, 6	1.54	0.05	0.88
Females mixed vs. power	17, 6	1.057	0.1	0.75

## Data Availability

The data used in this study and Python code in Jupyter notebook developed to perform computations are available via GitHub repository https://github.com/erinijapranckeviciene/ppolygenic (accessed on 8 May 2021).

## Abbreviations

The following abbreviations are used in this manuscript:

TGS	Total Genotype Score
LR	Logistic Regression
WF	Williams and Folland
